# Effects of conservation measures on crop diversity and their implications for climate-resilient livelihoods: the case of Rupa Lake Watershed in Nepal

**DOI:** 10.1007/s11629-020-6426-3

**Published:** 2022-04-13

**Authors:** Yun-li Bai, Chao Fu, Balaram Thapa, Ram Balladur Rana, Lin-xiu Zhang

**Affiliations:** 1 Key Laboratory of Ecosystem Network Observation and Modeling, Institute of Geographic Sciences and Natural Resources Research, Chinese Academy of Sciences, Beijing, 100101 China; 2 United Nations Environment Programme-International Ecosystem Management Partnership (UNEP-IEMP), Beijing, 100101 China; 3 Local Initiatives for Biodiversity, Research and Development (LI-BIRD), PO Box 324, Pokhara, 33700 Nepal

**Keywords:** Agrobiodiversity, Conservation measures, Crop species and varieties, Rupa Lake

## Abstract

Agrobiodiversity conservation is vital for achieving sustainability, but empirical studies on the effects of different practices or measures on crop diversity are rare. This study aims to estimate the effects of raising conservation awareness (RCA), building diversity blocks (BDB), and their combination on crop diversity among 240 randomly selected households surrounding the Rupa Lake Watershed in Nepal. Based on descriptive analysis and multiple regression models, the results indicate that the two single measures had no significant effect on the numbers of crop species and varieties grown by households in 2018. However, the combination of RCA and BDB had a significantly positive effect on the number of crop varieties, especially for grain and vegetable crops. Considering that these crops are essential in the daily lives of local people, the results indicate that a strategy that combines both awareness raising and on-farm conservation measures can generate higher crop diversity and better serve the climate-resilient livelihoods of people in mountainous areas.

## 1 Introduction

Ending hunger, achieving food security and improving nutrition and health, as highlighted in the United Nation 2030 Agenda for Sustainable Development, calls for the conservation and sustainable use of agrobiodiversity (Jacobsen et al. 2015; Zimmerer and de Haan 2017), which particularly needs strengthening amidst the coronavirus disease (COVID-19) crisis (Zimmerer and de Haan 2020). Agrobiodiversity refers to the diversity of plants, animals, and microorganisms that underpin agricultural systems (Wood and Lenné 1999; Narloch et al. 2013; Dedeurwaerdere and Hannachi 2019). Different studies have shown its importance in terms of supplying genetic resources that provide a wide range of critical benefits, such as income opportunities and nutritious diets (Kahane et al. 2013; Sibhatu and Matin 2018), the provision of ecosystem services such as soil health and water conservation (Hajjar et al. 2008), and adaptations to climate change in agricultural systems (Mijatovic et al. 2013; Dempewolf et al. 2014; Kozicka et al. 2020).

Agrobiodiversity is even more important for smallholders in mountainous areas. Compared with urban and plain areas, mountainous rural communities are more vulnerable in both ecological and socioeconomic aspects (Kruijssen et al. 2009). Agriculture is always the most important sector for employment and income sources for local populations in these areas, whereas they are also susceptible to frequent natural disasters, soil erosion and ecological degradation (Panagos et al. 2018). There is broad evidence that agrobiodiversity, especially crop diversity, which is the most valuable component of agrobiodiversity, can provide natural insurance to risk-averse farmers (Baumgärtner and Strunz 2009; Di Falco et al. 2010). With diversified crop species and varieties, farmers can adapt crops and their livelihoods to changing environments (Fowler and Hodgkin 2004). Crop diversity is thus very important for the functioning of both ecological and agricultural systems (Johns et al. 2013).

Over the last few decades, a range of practices or measures have become available to help farmers and communities conserve and utilize local crop genetic diversity in their farm systems (Jarvis et al. 2011). Recent evidence implies that crop diversification practices can contribute to climate-smart agriculture by improving productivity, livelihood outcomes, resilience of cropping systems and reducing carbon dioxide emissions (Makate et al. 2016), and thus is a promising strategy for farmers to adapt to climate change. However, few recommendations exist on how to diversify cropping systems in ways that best fit the agroecological and socioeconomic challenges farmers face (van Zonneveld et al. 2020). An important research question that remains largely uninvestigated is how to work out which measures would be the most relevant for crop diversity in a specific situation (Mzyece and Ng'ombe 2020).

Nepal is a mountainous least developed country located in the Himalayan region, a known hot spot of crop diversity and climate change (Agnihotri and Palni 2007; Yao et al. 2012; Sharma et al. 2019). Since the 1990s, increasing awareness about the importance of crop diversity has been driving efforts to enhance the conservation and utilization of crop diversity in Nepal (Gauchan et al. 2017; Joshi et al. 2017). Dozens of on-farm conservation methods and practices for different crops have been performed by local organizations, such as diversity blocks and raising conservation awareness (RCA) (Sthapit et al. 2012). Some studies have investigated the socioeconomic and institutional factors that influence farmers' decisions and measures to promote crop diversity in Nepal (Gauchan et al. 2005; Bragdon et al. 2009; Poudel and Johnsen 2009; Bhattarai et al. 2015). Further research is needed to quantitatively assess the effects of different measures on the on-farm conservation of local crop diversity.

The main objective of this study was to investigate the effects of two conservation measures on crop diversity, using the agro-ecosystems in Nepal's Rupa Lake Watershed (RLW) as a case study. The two measures are raising conservation awareness (RCA) and building diversity blocks (BDB) of grain crops, vegetables, cash crops, and other crops. Based on the results of this study, their implications for both crop diversity conservation and climate-resilient livelihoods in mountainous areas such as the RLW are also explored.

## 2 Materials and Methods

### 2.1 Context of the study site

The RLW is located in the Pokhara valley (28°08'10" to 28°12'24.4" N, 84°05'54.5" to 84°10'5.3" E), Kaski District of Gandaki Province, approximately 200 km west of Kathmandu, the capital city of Nepal (Fig. 1). The climate of the country is sub-tropical, humid, and marked by heavy monsoon rainfall (>2000 mm). However, the observed climatic trend analysis in the districts of Nepal (1971-2014) showed that the annual maximum temperature trend was positive (0.07-0.09°C per year) and significant, but the annual precipitation was significantly decreasing in Kaski (DHM 2017). The

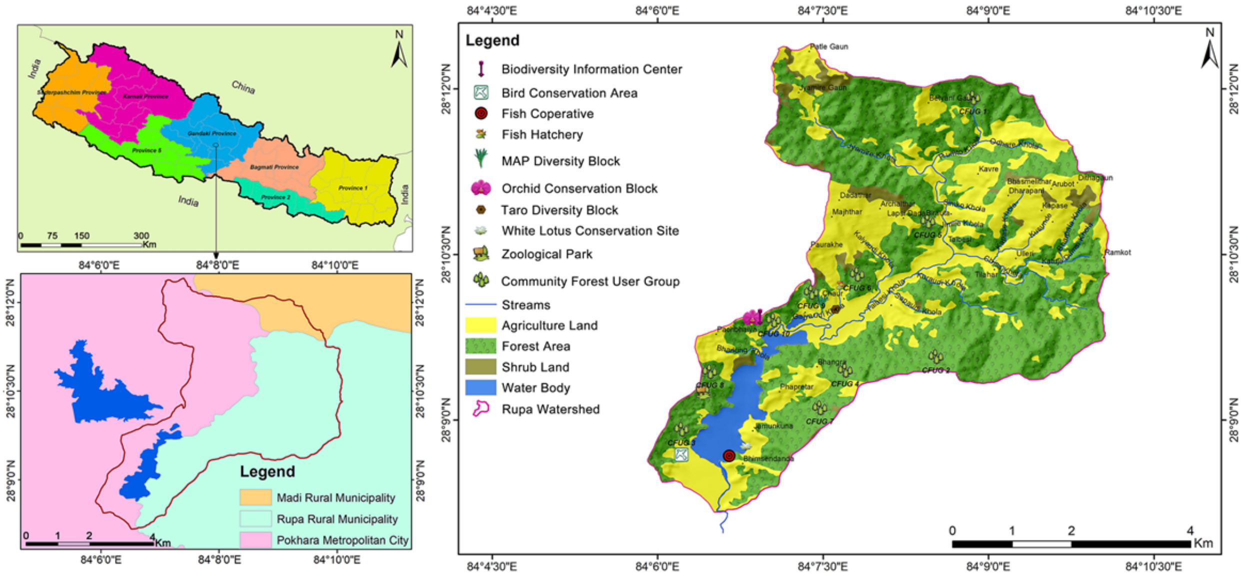

**Fig. 1** Location, landscape and resource sketch map of the Rupa Lake Watershed.

RLW covers a total area of 2,707 ha of land, with steep slopes of 35°-60° and altitudes varying from 580 to 1,420 meters above sea level. Of the total area, 33.57% is agricultural land, 61.85% is forestland, 3.69% is water bodies, and 0.89% is barren land. Some 1,933 households of various castes and ethnicities live in the watershed area. The watershed inhabitants have, for generations, depended on living on integrated farming systems in which farmland, forest, livestock, and water resources are intertwined. Historically, these ecosystems have experienced periods of deterioration due to unsustainable agricultural practices and the overuse of watershed resources, which have had negative impacts on the habitats of local plants, such as wild rice, white lotus, wetland birds and fish varieties (Chaudhary et al. 2015).

In the past two decades, several projects relevant to crop diversity conservation and watershed management were implemented in the RLW (Bogati 1996). The large majority of these projects were implemented through two local organizations, i.e., Jaibikshrot Samarachyan Abhiyan (JSA) and Rupa Lake Restoration and Fishery Cooperative (RLRFC), with support from national and international organizations such as the Local Initiatives for Biodiversity, Research and Development (LI-BIRD). A community-based management approach was adopted to empower local communities to restore, conserve and sustainably utilize crop diversity to obtain resilient livelihood outcomes (Sthapit and Mijatovic 2014). Several measures for conserving crop diversity were carried out in the RLW, notably RCA and BDB. Specifically, RCA comprises activities such as the promotion of diversity fairs and festivals, poetry journeys, Teej songs, community-led lake cleaning campaigns, plantation campaigns, community biodiversity registers, travelling seminars, and the establishment of biodiversity information centers. The diversity block is an experimental plot of farmers' varieties that is established and managed by local communities for research and development purposes (Sthapit et al. 2012). Building a functional diversity block needs to follow several steps and processes, such as collecting seed samples, field layout, planting and labelling the crops, harvesting seeds, and storing seeds. With initial external support, the BDB is used as a practical approach not only to demonstrate the total amount of local crop varieties and landraces (e.g. rice, taro, finger millet, orchid, wild rice, white lotus, and medicinal plants) at public places, but also to regenerate seeds and meet the seed demands for local varieties from community members.

## 2.2 Sample selection and data collection

From the total 1,933 households dwelling in the lake basin, 240 households were selected based on stratified random sampling method for face-to-face interviews with questionnaires in November-December 2019. Out of 240 sample households, 120 households (50%) were randomly selected from the list of shareholders in the RLRFC and member households of JSA, while the remaining households were non-shareholders of the RLRFC and nonmembers of JSA. The total number of households that were RLRFC shareholders and/or JSA members reached 1185.

Information on household characteristics, farmland endowment, and crop production in 2018 was collected from the sample households. Specifically, regarding household characteristics, we gathered information for each family member, including age, gender, years of schooling, and ethnicity. With such information, we could obtain household demographic characteristics, such as dependency, education of labourers, and ethnicity of the household. The information on farmland endowment included the types of land owned by a household and the area and number of plots of each type of land, such as paddy fields, dry farmland, orchards, tea gardens, forestland, grassland, and fish ponds.

Information on crop production included the names and numbers of species and the numbers of varieties, which were used to measure crop diversity. In addition, information on the interviewees' perception of climate-related disasters was collected during the household interview. All the data were cleaned and analysed by Stata 15 (StataCorp 2017), a professional software package for social and economic data analysis.

## 2.3 Analysis method

In this study, the sample households that participated in the same measure (RCA, BDB, or the two combined) were identified as a 'treatment' group, named after the measure they participated. For each treatment group, all the other sample households that did not participate in any measure were identified as the 'control' group.

We first used descriptive statistical analysis to capture the basic characteristics of crop diversity in the RLW and then conducted a T-test to determine the differences in crop diversity between the treatment and control groups.

The descriptive analysis results identified some intuitive relations between conservation measures and crop diversity. However, the latter was also subject to other factors, which may have been correlated with the measures and thus may have biased the results. Multivariate regressions were adopted to obtain more accurate results by controlling for confounding factors that were correlated with both conservation measures and crop diversity. Therefore, we further adopted multiple regression models (Cramer and Ridder 1991; Matejka and McKay 2015) to estimate the impact of conservation measures on the on-farm crop diversity managed by households. The specifications of the models are as follows:

$$\text{Crop}_i = \alpha_0 + \alpha_1 \text{Intervention}_i + \alpha_2 H_i + \alpha_3 L_i + \varepsilon_i \quad (1)$$

$$\text{Variety}_i = \alpha_0 + \alpha_1 \text{Intervention}_i + \alpha_2 H_i + \alpha_3 L_i + \alpha_4 C_i + \varepsilon_i \quad (2)$$

where $i$ indicates the $i$-th household in the equations.

$\text{Crop}_i$ is the vector of dependent variables in Eq. (1), including crop, staple, grain, cash, fruit, and vegetable. These variables indicate the total numbers of species of all the crops, staple grain crops, other grain crops, cash crops, fruits, and vegetables grown by a household in 2018, respectively (see all the variables in Table 1, similarly hereinafter). Staple grain crops include rice, wheat, corn, and potato. Other grain crops include soybean, beans, millet, barley, and cassava. Cash crops include peanut, oilseed rape, spices, tea, coffee, herbs, and so on.

$\text{Variety}_i$ is the vector of dependent variables in Eq. (2), including variety, staple_variety, grain_variety, cash_variety, fruit_variety, and vegetable_variety. These variables indicate the total numbers of varieties of all the crops, staple grain crops, other grain crops, cash crops, fruits, and vegetables grown by a household in 2018, respectively.

$\text{Intervention}_i$ is the vector of independent variables on the conservation measures in the equations. There are three specific variables, including RCA, BDB, and combined measures. The RCA and BDB indicate the measures mentioned in Section 2.1. The combined measures variable means that the joint measure of RCA and BDB is implemented by a household. In this study, households that intervened with combined measures were excluded from those that intervened with RCA or BDB to differentiate the effects of combined and single measures.

$H_i$ is the vector of the control variable on household characteristics, including the demographic structure and human capital of a household. The conservation and use of crop genetic diversity always involve labour-intensive activities such as local seed selection, savings and exchange (Su et al. 2016). The demographic structure measured by the dependency ratio is an indicator used to capture the labour situation and family care burden of a household. The

**Table 1** Description of variables

| Variables | Definition | Obs | Mean | Std. D. | Min | Max |
|---|---|---|---|---|---|---|
| RCA | Whether intervened with only raising conservation awareness, such as diversity fair and festival, poetry journeys, teej songs, or information centres (1=yes, 0=no) | 240 | 0.117 | 0.322 | 0 | 1 |
| BDB | Whether intervened with only building diversity blocks of rice, taro, finger millet, orchid, wildrice, white lotus, or medicinal plants (1=yes, 0=no) | 240 | 0.088 | 0.283 | 0 | 1 |
| Combined measures | Whether intervened with both conservation awareness and diversity blocks (1=yes, 0=no) | 240 | 0.150 | 0.358 | 0 | 1 |
| Crop | Total number of crop species | 240 | 5.933 | 2.042 | 1 | 12 |
| Staple | Number of staple grain crops | 240 | 2.075 | 0.860 | 0 | 4 |
| Grain | Number of other grain crops | 240 | 1.025 | 0.914 | 0 | 3 |
| Cash | Number of cash crops | 240 | 1.183 | 0.858 | 0 | 4 |
| Fruit | Number of fruits | 240 | 0.658 | 0.556 | 0 | 3 |
| Vegetable | Whether plant vegetable (1=yes, 0-no) | 240 | 0.954 | 0.210 | 0 | 1 |
| Variety | Total number of varieties | 240 | 17.251 | 7.416 | 0 | 38 |
| Staple_variety | Number of varieties of staple grain crops | 240 | 3.492 | 1.994 | 0 | 12 |
| Grain_variety | Number of varieties of other grain crops | 240 | 1.438 | 1.596 | 0 | 9 |
| Cash_variety | Number of varieties of cash crops | 240 | 3.289 | 2.698 | 0 | 13 |
| Fruit_variety | Number of varieties of fruits | 240 | 1.658 | 2.453 | 0 | 20 |
| Vegetable_variety | Number of varieties of vegetables | 240 | 7.617 | 4.481 | 0 | 40 |
| Land | Land size (ha) | 240 | 0.434 | 0.330 | 0 | 2.15 |
| Land_staple | Land size of paddy field and dry farmland (ha) | 240 | 0.423 | 0.326 | 0 | 2.15 |
| Land_grain | Land size of dry farmland (ha) | 240 | 0.184 | 0.185 | 0 | 1.25 |
| Land_cash | Land size of dry farmland, orchards, and tea gardens (ha) | 240 | 0.195 | 0.192 | 0 | 1.25 |
| Land_fruit | Land size of orchards and tea gardens (ha) | 240 | 0.011 | 0.062 | 0 | 0.9 |
| Dependency | Dependency ratio in a household (%) | 239 | 44.159 | 47.154 | 0 | 300 |
| Education | Average years of schooling of labours in household | 239 | 7.664 | 2.715 | 0 | 15 |
| Ethnicity | Whether belong to the BCT (Bahun, Chhetri, Tharu) ethnic groups (1=yes, 0=no) | 240 | 0.788 | 0.410 | 0 | 1 |
| Drought | Whether the interviewee think the times of drought decreased compared with five years ago (1=yes, 0=no) | 240 | 0.054 | 0.227 | 0 | 1 |
| Flood | Whether the interviewee think the times of flood decreased compared with five years ago (1=yes, 0=no) | 240 | 0.546 | 0.499 | 0 | 1 |
| Landslide | Whether the interviewee think the times of landslide decreased compared with five years ago (1=yes, 0=no) | 240 | 0.313 | 0.464 | 0 | 1 |
| Gender_interviewee | Gender of interviewee (1=male, 0=female) | 240 | 0.425 | 0.495 | 0 | 1 |
| Age_interviewee | Age of interviewee (years) | 240 | 49.550 | 13.390 | 21 | 70 |
| Education_interviewee | Years of schooling of the interviewee (years) | 240 | 8.871 | 4.252 | 0 | 22 |
| Ethnicity_interviewee | Whether the interviewee belong to the BCT (Bahun, Chhetri, Tharu) ethnic groups (1=yes, 0=no) | 240 | 0.783 | 0.413 | 0 | 1 |

**Note:** Due to missing data, the sample size of (21) dependency and (22) education is 239.

human capital measured by average years of schooling of laborers in a household plays an important role in making decisions on crop planting. Ethnic culture in the form of rituals, food traditions and religious practices provides incentives or norms for the maintenance of local traditional crop diversity (Subedi et al. 2011; Negi and Maikhuri 2013). Therefore, $H_i$ has three variables of *dependency, education, and ethnicity*, which have been controlled in other similar studies (Chen and Meng 2007).

$L_i$ is the vector of the control variable on land endowment which is measured by the landholding size. $C_i$ in Eq. (2) is the variable of *crop species*, which is used to control the effect of crop species on the varieties. $\alpha_i$ ($i$=0, 1, 2, 3, 4) is the vector of the coefficients that captures the determinants of crop diversity, in which $\alpha_1$ is the effect of conservation measures on the dependent variables. $\alpha_0$ is the constant term, and $\varepsilon_i$ is the error term.

During the interview, approximately 30%, 20%, and 48% of households did not farm other grain crops, cash crops, and fruits in 2018, respectively. Therefore, there were censored data of the dependent variables of grain, cash, fruit, grain_variety, cash_variety, and fruit_variety. For the continuous dependent variables, we used the ordinary least squares (OLS) to conduct parameter estimation. For the censored dependent variables, we adopted maximum likelihood estimation (MLE) for Tobit regressions. For the binary dependent variables, we also adopted MLE for Probit regressions.

## 3 Results

### 3.1 Crop diversity in the RLW

The households in the RLW grew 5.933 crop species in 2018 on average. Among these, staple grain crops accounted for nearly 35% and more than 2 species. Additionally, they grew 1.025, 1.183, and 0.658 species of other grain crops, cash crops, and fruits, respectively. Most (95.4%) households grew vegetables.

An average of 17.251 crop varieties were grown by the households in the RLW, including 3.492 varieties of staple grain crops, 1.438 varieties of other grain crops, and 1.658 varieties of fruits. Cash crops were important income sources and were more likely to be grown in this area. The average number of cash crop varieties grown by these households was 3.289, second only to staple grain crops. Vegetables are very important for diversified foods in mountainous areas, especially in remote and less developed rural areas. An average of 7.617 vegetable varieties was grown among these households, much more than that of any other crop category.

### 3.2 Crop diversity under different measures

There were 11.67%, 8.75%, and 15% of households in the RLW participating in the RCA, BDB, and combined measures treatment groups, respectively. Most households (64.58%) were not intervened with any measure and were thus classified in the control group.

There were some differences in crop species diversity between the RCA treatment and control groups, but the differences were not statistically significant. The average number of crop species grown by the RCA treatment group was 5.679, which was 0.360 less than that grown by the control group (Table 2, row 1). The average numbers of staple and other grain crop species grown by the RCA treatment group were 0.049 more and 0.242 less than those grown by the control group, respectively (Table 2, rows 2 and 3). The cash crop species and fruit species grown by the RCA treatment group were not as diversified as those grown by the control group (Table 2, rows 4 and 5). However, the households of the RCA treatment group were more likely to plant vegetables (Table 2, row 6).

The results of the descriptive analysis indicated some significant differences in crop species diversity between the BDB treatment and control groups. The average number of all crop species grown by the BDB

**Table 2** Descriptive analysis of crop species diversity under different conservation measures

| Variables | | | Mean | | Difference =(2) – (1) | T-test | *p*-value |
|---|---|---|---|---|---|---|---|
| | | | (1) Yes | (2) No | | | |
| (1) | RCA | Crop | 5.679 | 6.039 | 0.360 | 0.878 | 0.381 |
| (2) | | Staple | 2.107 | 2.058 | -0.049 | -0.292 | 0.771 |
| (3) | | Grain | 0.893 | 1.135 | 0.242 | 1.321 | 0.188 |
| (4) | | Cash | 1.071 | 1.200 | 0.129 | 0.726 | 0.449 |
| (5) | | Fruit | 0.607 | 0.665 | 0.057 | 0.480 | 0.632 |
| (6) | | Vegetable | 0.964 | 0.961 | -0.003 | -0.076 | 0.939 |
| (7) | BDB | Crop | 5.095 | 6.039 | 0.943 | 1.982 | 0.049** |
| (8) | | Staple | 2.048 | 2.058 | 0.010 | 0.053 | 0.958 |
| (9) | | Grain | 0.476 | 1.135 | 0.659 | 3.214 | 0.002*** |
| (10) | | Cash | 0.905 | 1.200 | 0.295 | 1.488 | 0.138 |
| (11) | | Fruit | 0.619 | 0.665 | 0.045 | 0.334 | 0.739 |
| (12) | | Vegetable | 0.952 | 0.961 | 0.009 | 0.195 | 0.846 |
| (13) | Combined measures | Crop | 6.057 | 6.039 | -0.018 | -0.049 | 0.961 |
| (14) | | Staple | 2.143 | 2.058 | -0.085 | -0.527 | 0.599 |
| (15) | | Grain | 0.914 | 1.135 | 0.221 | 1.302 | 0.195 |
| (16) | | Cash | 1.314 | 1.200 | -0.114 | -0.722 | 0.471 |
| (17) | | Fruit | 0.686 | 0.665 | -0.021 | -0.197 | 0.844 |
| (18) | | Vegetable | 0.914 | 0.961 | 0.047 | 1.181 | 0.239 |

**Notes:** *** and ** indicate $p<0.01$, $p<0.05$, respectively. RCA is abbreviated for raising conservation awareness. BDB is abbreviated for building diversity blocks.

treatment group was significantly less than that grown by the control group ($p$<0.05) (Table 2, row 7). The differences in staple crop species diversity between the BDB treatment and control groups were not significant (Table 2, row 8). The average number of other grain crop species grown by the BDB treatment group was significantly less than that grown by the control group ($p$<0.01) (Table 2, row 9). The differences in cash crop and fruit species diversity between the BDB treatment and control groups were not significant (Table 2, rows 10 and 11). The households in the BDB treatment group were less likely to plant vegetables (Table 2, row 12). This result indicates that the differences in crop species diversity between the BDB treatment and control groups were mainly attributed to their differences in the diversity of other grain crop species.

The differences in crop species diversity between the treatment and control groups were minor and not significant even at the 10% level in the cases of combined measures. The average number of crop species in the combined measures group was 6.057, which was 0.018 more than that in the control group (Table 2, row 13). The average numbers of staple and other grain crop species in the combined measures group were 0.085 more and 0.221 less than those in the control group, respectively (Table 2, rows 14 and 15). The average numbers of cash crop species and fruit species in the combined measures group were more diversified than those in the control group (Table 2, rows 16 and 17). The probability of planting vegetables among the household figs in the combined measures group was lower than that in the control group (Table 2, row 18).

For crop variety diversity, the descriptive analysis showed some differences between the RCA and control groups. In terms of the average total number of varieties, there was no significant difference between the two groups. However, the average number of staple crop varieties in the RCA treatment group was significantly greater than that in the control group ($p$<0.1) (Table 3, row 2). The numbers of other grain crop, cash crop, fruit, and vegetable varieties in the RCA treatment group were less than those in the control group, but the differences were not significant (Table 3, rows 3-6).

All the average numbers of varieties in the BDB treatment group were less than those in the control group. However, the differences in the numbers of staple crop, cash crop, fruit, and vegetable varieties between the two groups were not significant even at the 10% level (Table 3, rows 8, 10, 11, and 12). The average number of other grain crop varieties grown by the BDB treatment group was significantly less than that grown by the control group ($p$<0.01) (Table 3, row 9).

The combined measures seemed to have more effects on improving the variety diversity of all the crops and of the staple grain crops. The average number of all the crop varieties was significantly higher than that in the control group ($p$<0.05) (Table 3, row 13). The staple crop varieties grown by the

**Table 3** Descriptive analysis of crop variety diversity under different conservation measures

| Variables | | | Mean | | Difference | T-test | $p$-value |
|---|---|---|---|---|---|---|---|
| | | | (1) Yes | (2) No | =(2) – (1) | | |
| (1) | RCA | Variety | 16.036 | 17.187 | 1.151 | 0.758 | 0.449 |
| (2) | | Staple_variety | 3.857 | 3.187 | -0.670 | -1.877 | 0.062* |
| (3) | | Grain_variety | 1.286 | 1.516 | 0.230 | 0.723 | 0.471 |
| (4) | | Cash_variety | 2.893 | 3.394 | 0.501 | 0.937 | 0.350 |
| (5) | | Fruit_variety | 1.429 | 1.645 | 0.217 | 0.456 | 0.649 |
| (6) | | Vegetable_variety | 6.571 | 7.439 | 0.867 | 1.053 | 0.294 |
| (7) | BDB | Variety | 14.81 | 17.187 | 2.378 | 1.407 | 0.161 |
| (8) | | Staple_variety | 3.143 | 3.187 | 0.044 | 0.113 | 0.910 |
| (9) | | Grain_variety | 0.571 | 1.516 | 0.945 | 2.735 | 0.007*** |
| (10) | | Cash_variety | 2.619 | 3.394 | 0.775 | 1.278 | 0.203 |
| (11) | | Fruit_variety | 1.190 | 1.645 | 0.455 | 0.844 | 0.400 |
| (12) | | Vegetable_variety | 7.286 | 7.439 | 0.153 | 0.164 | 0.870 |
| (13) | Combined measures | Variety | 19.971 | 17.187 | -2.784 | -2.009 | 0.046** |
| (14) | | Staple_variety | 4.657 | 3.187 | -1.470 | -4.038 | 0.000*** |
| (15) | | Grain_variety | 1.571 | 1.516 | -0.055 | -0.186 | 0.853 |
| (16) | | Cash_variety | 3.543 | 3.394 | -0.149 | -0.292 | 0.771 |
| (17) | | Fruit_variety | 1.657 | 1.645 | -0.012 | -0.028 | 0.978 |
| (18) | | Vegetable_variety | 8.514 | 7.439 | -1.076 | -1.414 | 0.159 |

**Notes:** *** and ** indicate $p$<0.01, $p$<0.05, respectively. RCA is abbreviated for raising conservation awareness. BDB is abbreviated for building diversity blocks.

households with combined measures were also significantly greater than those grown by the control group ($p$<0.01) (Table 3, row 14). The varieties of other grain crops, cash crops, fruits, and vegetables in the combined measures group were greater than those in the control group, but the differences were not significant (Table 3, rows 15-18).

## 3.3 Results of multivariate regressions

### 3.3.1 Effects of measures on crop species diversity

Consistent with the descriptive analysis results, the regression results showed that neither RCA nor the combined measures had a significant effect on crop species diversity since their coefficients were not significant even at the 10% level (Table 4, rows 1 and 3). The measure of BDB had a significantly negative effect on crop species diversity, especially on the diversity of other grain crop species (Table 4, row 2, columns 1, and 3).

Furthermore, as a critical variable of household characteristics, the dependency ratio had no significant effect on the diversity of most crop species except for significantly negative effects on cash crop and vegetable species diversity in this study ($p$<0.01, Table 4, row 4, columns 4 and 6). If the dependency ratio increased by 1%, the number of cash crop species decreased by 0.001, and the probability of growing vegetables in a household decreased by 0.1%. Growing cash crops and vegetables is always more labor-intensive than growing other categories of crops. A high dependency ratio means there are more elderly individuals and children in the household, which implies there is no enough labor to be engaged in growing cash crops and vegetables.

Farmland endowment was another important factor affecting crop species diversity. If the landholding size increased by 1 ha, the total number of crop species increased by approximately 1.431 ($p$<0.01, Table 4, row 7, column 1). The numbers of staple and cash crop species were more likely to be affected by landholding size ($p$<0.01, Table 4, rows 8 and 10, columns 2 and 4). Previous studies found that maintaining high crop diversity might be hard for small landholders because of their low-quality land, and they could rarely grow multiple crops (Isakson 2011; McDougall et al. 2013). The quality of land used

**Table 4** Impacts of interventions on the number of crop species

| Variables | | Crop (1) | Staple (2) | Grains (3) | Cash (4) | Fruit (5) | Vegetable (6) |
|---|---|---|---|---|---|---|---|
| (1) | RCA | -0.486 (0.412) | 0.006 (0.176) | -0.187 (0.134) | -0.095 (0.122) | -0.025 (0.077) | -0.006 (0.041) |
| (2) | BDB | -0.819* (0.464) | 0.033 (0.198) | -0.517*** (0.120) | -0.162 (0.133) | -0.028 (0.086) | -0.013 (0.046) |
| (3) | Combined measures | -0.061 (0.378) | 0.037 (0.161) | -0.128 (0.128) | 0.051 (0.120) | -0.002 (0.073) | -0.037 (0.037) |
| (4) | Dependency | -0.002 (0.003) | -0.001 (0.001) | 0.001 (0.001) | -0.001* (0.001) | 0.001 (0.001) | -0.001** (0.000) |
| (5) | Education | 0.046 (0.049) | 0.017 (0.021) | 0.011 (0.017) | -0.007 (0.015) | 0.006 (0.009) | 0.009* (0.005) |
| (6) | Ethnicity | 0.131 (0.327) | 0.104 (0.139) | -0.054 (0.110) | 0.094 (0.099) | 0.063 (0.061) | -0.014 (0.032) |
| (7) | Land | 1.431*** (0.409) | | | | | |
| (8) | Land_staple | | 0.501** (0.176) | | | | |
| (9) | Land_Grain | | | 0.167 (0.248) | | | 0.045 (0.070) |
| (10) | Land_cash | | | | 0.859*** (0.210) | | |
| (11) | Land_fruit | | | | | 0.614 (0.401) | |
| (12) | Constant | 5.101*** (0.473) | 1.713*** (0.202) | | | | 0.929*** (0.047) |
| (13) | Observations | 239 | 239 | 239 | 239 | 239 | 239 |

**Note:** Standard errors in parentheses. *** indicates $p$<0.01, ** indicates $p$<0.05, * indicates $p$<0.1.
The marginal effects are reported in columns (3), (4), and (5).

for growing staple and cash crops was usually higher than that of land used for growing other crops. Therefore, the coefficients of landholding size and crop species number were significant in the cases of staple grain and cash crops in this study. The numbers of other grain crop and fruit species were not affected by landholding size. For many households, other grain crops, such as beans, were usually consumed by the households themselves and thus planted in small areas or even interplanted with staple grain crops. Fruit trees were also planted in small areas and even on barren land. Therefore, their species numbers were not sensitive to landholding size.

### 3.3.2 Effects of measures on crop variety diversity

Looking at the effects of measures after controlling other variables, we found that BDB had no significant effect on the varieties even at the 10% level (Table 5, row 2). The measure of RCA had a positive effect on the number of varieties of staple grain crops (Table 5, row 1, column 2). The combined measures had significant effects on crop variety diversity (Table 5, row 3). Specifically, the average number of all crop varieties grown by the combined measures treatment group was 4.102 more than that grown by the control group ($p$<0.01, Table 5, row 3, column 1). The average numbers of varieties of staple grain crops, other grain

**Table 5** Impacts of interventions on the number of crop varieties

| Variables | | Variety (1) | Staple_variety (2) | Grains_variety (3) | Cash_variety (4) | Fruit_variety (5) | Vegetable_variety (6) |
|---|---|---|---|---|---|---|---|
| (1) | RCA | -0.603 (1.566) | 0.579** (0.288) | 0.051 (0.152) | -0.138 (0.337) | -0.031 (0.241) | -0.807 (0.836) |
| (2) | BDB | 0.080 (1.771) | 0.043 (0.324) | -0.181 (0.190) | -0.012 (0.390) | -0.179 (0.257) | -0.014 (0.946) |
| (3) | Combined measures | 4.102** (1.434) | 1.375*** (0.263) | 0.269* (0.146) | 0.177 (0.325) | 0.266 (0.243) | 2.164** (0.766) |
| (4) | Dependency | -0.008 (0.010) | -0.002 (0.002) | -0.001 (0.001) | 0.001 (0.002) | -0.001 (0.002) | 0.002 (0.006) |
| (5) | Education | 0.250 (0.185) | -0.018 (0.034) | 0.005 (0.018) | 0.034 (0.041) | -0.003 (0.029) | 0.150 (0.101) |
| (6) | Ethnicity | 0.471 (1.238) | 0.292 (0.228) | -0.012 (0.115) | -0.114 (0.269) | 0.186 (0.191) | 0.617 (0.651) |
| (7) | Crop | 2.377*** (0.249) | | | | | |
| (8) | Staple | | 1.496*** (0.108) | | | | |
| (9) | Grains | | | 1.134*** (0.052) | | | |
| (10) | Cash | | | | 1.775*** (0.131) | | |
| (11) | Fruit | | | | | 1.371*** (0.148) | |
| (12) | Vegetable | | | | | | 7.738*** (1.345) |
| (13) | Land | 3.436** (1.589) | | | | | |
| (14) | Land_staple | | 0.426 (0.292) | | | | |
| (15) | Land_grain | | | 0.494* (0.267) | | | 3.345** (1.439) |
| (16) | Land_cash | | | | 0.967 (0.593) | | |
| (17) | Land_fruit | | | | | 0.100 (1.265) | |
| (18) | Constant | -0.479 (2.197) | -0.054 (0.378) | | | | -2.328 (1.578) |
| (19) | Observations | 239 | 239 | 239 | 239 | 239 | 239 |

**Note:** Standard errors in parentheses. *** indicates $p$<0.01, ** indicates $p$<0.05, * indicates $p$<0.1. The marginal effects are reported in columns (3), (4), and (5).

crops, and vegetables grown by the combined measures treatment group were 1.375, 0.268 and 2.164, respectively, more than those grown by the control group ($p<0.05$, Table 5, row 3, columns 2, 3 and 6). The results indicate that staple grain crops and vegetables contributed the most to the increase in the total number of crop varieties grown by households under combined measures.

In contrast to household characteristics, crop species significantly affected crop variety diversity in this study. The regression results show that no household characteristics were related to crop variety diversity (Table 5, rows 4, 5 and 6). However, the types of crop species were highly related to crop variety diversity. Specifically, the coefficient of the variable *crop* was 2.377, which implies that the number of crop varieties would increase by 2.377 following an increase of 1 in the number of crop species ($p<0.01$, Table 5, row 7, column 1). The coefficients for different categories of crops were all over 1 ($p<0.01$, Table 5, rows 8-12, columns 2-6), which indicated that crop variety diversity expanded more dramatically than did crop species diversity. A reason to explain the result is that, given that climatic impacts vary less among different varieties of a specific crop species than among different crop species (Raza et al. 2019), farmers are inclined to adopt multiple varieties to reduce climate-related farming risks.

Farmland endowment also had significant effects on crop variety diversity. 1-ha increase of land would result in increase of 3.436 varieties ($p<0.01$, Table 5, row 13, column 1). The impact of land endowment on crop variety diversity differed among categories of crop species. For staple grain crops, the expansion of landholding size did not lead to a corresponding increase in the number of crop varieties (Table 5, row 14, column 2), which implies the trend of specialized production of grain crops along landholding size enlargement. However, for other grain crops and vegetables, 1-ha increase of land would lead to increase of 0.494 and 3.345 varieties, respectively ($p<0.1$, Table 5, row 15, columns 3 and 6). Other grain crops are considered as livelihood crops due to their plenty of vegetable proteins and are usually planted for traditional festivals. Vegetables are high-yield, whereas also high-risk in climate shocks, thus farmers may tend to grow more diversified crops to lower risk and stabilize crop income (Khan and Verma 2018).

## 4 Discussion

The results of this study have evident implications for both crop diversity conservation and climate-resilient livelihoods in mountainous areas like the RLW. In terms of crop diversity conservation, neither of the two single measures but their combination could increase the crop diversity of households in the RLW. The results indicate at least two things. First, there is a considerable challenge in enhancing crop diversity that is demanding on a range of local climatic, geographical, and socioeconomic regimes (Raza et al. 2019; Kozicka et al. 2020). Second, a comprehensive strategy that brings farmers multiple kinds of support, from knowledge to material sources, can help them address such a challenge more effectively. It can be expected that introducing more conservation measures, e.g. capacity building through training and workshops, product value addition and marketing, will enhance the effects of existing measures on crop diversity.

This study demonstrated the potential mechanisms underlying different measures for conserving crop diversity. RCA activities are essential for exposing farming communities to the knowledge and information on biodiversity, and for inspiring them to participate in the conservation and management of crop diversity (Shrestha et al. 2013). However, most of them do not directly translate into the adoption new varieties if without access of seeds and other materials. In contrast, diversity blocks can provide a constant supply of seeds. However, the success and sustainability of BDB is dependent upon the interest, level of awareness, and capacity of the local community in sustaining the varieties and seed sources, especially given they need to address major technical and financial problems in linking diversity blocks with community seed production, community seed banks and local markets. We thus inferred that the BDB measure when combined with RCA activities can increase both access to seeds and knowledge and thus be effective in enhancing crop diversity.

In terms of implications for livelihoods, it was found in this study that combined measures had significantly positive effects on the variety diversity of staple grain crops, other grain crops and vegetables. Staple grain crops are the most essential crops needed in daily life, while other grain crops and vegetables are the main sources of multiple nutrients that are critical for human health, especially for smallholder farmers in mountainous areas, as these farmers are poor and have little market access. Although cash crops and fruits can make more profits than other crops, they always require increased inputs and face more technical and environmental risks in production. Our results support the assumption that crop diversity conservation can be enhanced by focusing on small-scale and low-input production processes (Johns and Sthapit 2004).

Previous studies argued that a higher diversity of crops could strengthen farmers' adaptive capacity and resilience, allowing for increased productivity, stable incomes and nutritional security at household level (Pellegrini and Tasciotti 2014; Makate et al. 2016; Kozicka et al. 2020). In this study, we did not investigate household livelihoods, and thus could not assess livelihood outcomes of conservation measures in the study area. Instead, our results from a multivariate regression indicated that the total number of crop varieties had a positive effect on the interviewee's perception of decreasing climate-related disasters compared with five years ago (Table 6, row 1). We preliminarily inferred combined measures that deliver higher crop diversity can support the livelihoods of smallholder farmers and rural communities by alleviating the impacts of climate-related disasters. However, the effects of different conservation measures on smallholder farmers' livelihood resilience need to be further investigated under an extended framework for vulnerability assessment combined with a sustainable livelihood framework (Xu et al. 2020).

Additionally, our results indicated significant effects of farmland endowment measured by landholding size on crop diversity in the RLW. A previous survey in the same area indicated that landholding size was one of the most important factors influencing the willingness of farmers to pay for rice landrace conservation (Poudel and Johnsen 2009). This study provides evidence of an increase in crop diversity with land endowment.

## 5 Conclusion and Perspectives

Our results showed that combined measures significantly improved crop variety diversity, especially for staple grain crops, other grain crops, and vegetables. This result indicates that the combined measures are an efficient way to protect crop variety diversity, which is the core of agrobiodiversity. We inferred that the BDB measure when combined with RCA activities can increase both access to seeds and knowledge that are needed to sustain and enhance crop diversity.

Grains and vegetables are both essential to maintain the food security, nutrition and health of farmers in mountainous areas. From this perspective, a combination of both raising awareness and on-farm conservation measures is recommended to generate higher crop variety diversity and improve the livelihoods of people in other mountainous areas. Considering the significant effect of farmland endowment on both crop species and variety diversities, innovating land use mechanisms to improve farmland endowment for smallholder farmers may be a promising means to promote crop diversity.

This study has identified several determinants of crop diversity among smallholder farmers in mountainous areas using local household survey data, which may be a valuable addition to the existing literature. However, we are fully aware of some shortcomings in this study. For example, due to a limited sample size, estimations of the heterogeneous effects of conservation measures among different groups of households were lacking. For the same reason, we did not analyse the impact of different

**Table 6** Impacts of crop varieties on the interviewee's perception of climate change

| Variables | Whether the interviewee think the disaster decreasing compared with five years ago (1=yes, 0=no) | | |
|---|---|---|---|
| | Drought | Flood | Landslide |
| Variety | 0.003** (0.001) | 0.010* (0.005) | 0.012*** (0.005) |
| Crop | -0.013 (0.008) | -0.019 (0.019) | -0.014 (0.018) |
| Land | -0.129** (0.055) | -0.038 (0.104) | 0.080 (0.092) |
| Gender_interviewee | -0.049 (0.031) | 0.164** (0.077) | -0.134* (0.070) |
| Age_interviewee | 0.002* (0.001) | 0.003 (0.003) | 0.010*** (0.003) |
| Ethnicity_interviewee | -0.026 (0.030) | 0.061 (0.078) | -0.166** (0.068) |
| Education_interviewee | 0.008** (0.004) | 0.004 (0.010) | 0.021** (0.008) |
| Observations | 240 | 240 | 240 |

**Note:** Standard errors in parentheses. *** indicates $p$<0.01, ** indicates $p$<0.05, * indicates $p$<0.1.

RCA activities and could not suggest specific activities that should be promoted in combined measures. It should be noted that some RCA activities, such as diversity fairs, are not only a participatory tool for raising public awareness on the value of conserving local varieties, but also provide an opportunity for the exchange of seeds and knowledge. These activities are more likely to increase levels of diversity in the farms of the participants when combined with the BDB measure. Finally, other measures beyond RCA and BDB should be addressed by further research in the future.

## Acknowledgments

The research reported in this manuscript was funded by the Natural Science Foundation of China (Grants No. 42061144004) and the Strategic Priority Research Program of Chinese Academy of Science (Grant No. XDA20010303).

## Open Access

This article is licensed under a Creative Commons Attribution 4.0 International License, which permits use, sharing, adaptation, distribution and reproduction in any medium or format, as long as you give appropriate credit to the original author(s) and the source, provide a link to the Creative Commons license, and indicate if changes were made. The images or other third party material in this article are included in the article's Creative Commons license, unless indicated otherwise in a credit line to the material. If material is not included in the article's Creative Commons license and your intended use is not permitted by statutory regulation or exceeds the permitted use, you will need to obtain permission directly from the copyright holder. To view a copy of this license, visit
http://creativecommons.org/licenses/by/4.0/.
